# Orthorexia nervosa: Research based on invalid measures is invalid

**DOI:** 10.7189/jogh.14.03007

**Published:** 2024-02-02

**Authors:** Juan Ramón Barrada, Adrian Meule

**Affiliations:** 1 Department of Psychology and Sociology, University of Zaragoza, Zaragoza, Spain; 2 Department of Psychiatry and Psychotherapy, LMU University Hospital, LMU Munich, Munich, Germany; 3 Schoen Clinic Roseneck, Prien am Chiemsee, Germany; 4 Institute of Medical Psychology, Faculty of Medicine, LMU Munich, Munich, Germany



Orthorexia nervosa (ON) refers to a strong preoccupation with a healthy diet with negative emotional, cognitive, or social consequences when trying to approach this goal and when the eating behaviour deviates from these self-imposed rules. According to a recent meta-analysis published in this journal, the prevalence of ON could be as high as 27.5% [1]. If that was true, ON – which is not (yet) a recognised eating disorder in diagnostic classification systems – would be the most prevalent of all eating disorders and, in fact, of all mental disorders [2]. There are two explanations for this. Either humans have been unaware of this eating disorder pandemic or the results from that meta-analysis suffer from a systematic bias in measurement. We consider the second one more likely.

Most importantly, the data that the meta-analysis is based on all came from studies that used the ORTO-15 questionnaire [3]. However, recent evidence shows this scale produces invalid scores for assessing ON [4]. Even the meta-analysis’s authors acknowledge the ORTO-15’s limitations in the discussion section. Given that ‘validity refers to the degree to which evidence and theory support the interpretations of test scores for proposed uses of tests’ (p. 11, [5]), no validity implies that the scores cannot be interpreted as intended. More clearly stated, the ORTO-15 does not measure ON and should not be interpreted as doing so. The logical conclusion is that any study using it (including meta-analyses) adds very limited knowledge to the scientific record or, even worse, it can be considered noise that some people will interpret as evidence. It has to be remembered that ‘validity is, therefore, the most fundamental consideration in developing tests and evaluating tests’ (p. 11, [5]).

## PROBLEMS IN THE ASSESSMENT OF ORTHOREXIA NERVOSA

Unfortunately, research on ON has been hampered for years due to the psychometric limitations of several of its measures. For example, one of the most prominent limitations of the ORTO-15 is its low internal reliability and absence of a first- or second-order one-factor structure [4]. Without this, item responses must not be summed to a total score, that is, computing total scores leads to uninterpretable values.

Other important limitations include the content of the items of the ORTO-15 but also of other commonly used instruments. Specifically, some items are either unspecific to ON (e.g., include no clear reference to healthy eating) or pathologise a healthy or neutral approach to eating. For unspecific items, consider ‘In the last three months, did the thought of food worry you?’ from the ORTO-15 or ‘Health professionals have expressed concern that my diet is too restrictive’ from the Orthorexia Nervosa Inventory [6]. These items probably tap relevant aspects of other eating disorders but not ON. For the tendency to pathologise healthy or neutral eating approaches, consider ‘Are you willing to spend more money to have healthier food?’ from the ORTO-15 or ‘Preparing food in the most healthful way is very important in my diet’ from the Orthorexia Nervosa Inventory. Considering this, the content validity of prominent questionnaires – not just the ORTO-15 – is uncertain.

## DEFINING AND DIAGNOSING ORTHOREXIA NERVOSA

Another important issue pertains to deriving meaningful cut-off scores that indicate the likely presence of ON and with which ON prevalences can be estimated. The problem is that – as ON is not an established eating disorder – there is no gold standard (e.g. a structured clinical interview) with which a diagnosis of ON could be established and that, therefore, could be used as a criterion variable. Thus, whether any dichotomisation of ON questionnaire scores is accurate must be questioned. Perhaps a dimensional approach (using questionnaire scores as a continuous variable) instead of a categorical one should be prioritised at this stage of knowledge about this condition.

This has also led to problems in theorising about ON. For instance, several meta-analyses about ON have been published before a consensus had been reached about a definition of this potentially new disorder and how to measure or diagnose it. Recently – striving to offer a big push to this area – a consensus document about the definition and diagnostic criteria for ON has been published [7]. Yet, we consider that what this document adds to the current knowledge about ON is limited. For example, when checking the references cited in that consensus document, it can be noted that the ORTO-15 was the primarily used questionnaire in a major part of the research that supports the statements made in that article. In line with the overall theme of this commentary, this consensus is, thus, at least partially based on research that likely suffers from a measurement bias.

## DISTINCTIVENESS FROM ANOREXIA NERVOSA

The lack of solid evidence surrounding ON has led some authors to question whether ON can be distinguished from other eating disorders [8]. For instance, Bhattacharya et al. [9] have argued that ON is a ‘new cultural manifestation’ of anorexia nervosa. While there seems to be a significant overlap between ON and anorexia nervosa (i.e. persons with anorexia nervosa have high scores on existing ON measures), this may also be due to the shortcomings of existing measures (e.g. because of the inclusion of unspecific items described above). Moreover, for an anorexia nervosa diagnosis, a requirement is a body weight that is less than minimally normal or – for children and adolescents – less than that minimally expected [2]. Yet, the association between scores on existing ON measures and body mass index is close to zero in samples from the general population, indicating that people with ON are not necessarily underweight [10]. Thus, the overlap with and distinctiveness from anorexia nervosa, and particularly from persons with atypical anorexia nervosa – who usually are also not underweight – is currently unclear and requires further examination.

## CONCLUSIONS

The current stage of research and knowledge about ON leaves more doubts than certainties. Among the certainties are: a) it is still very uncertain whether the prevalence of ON is almost ‘three out of ten’ [1], b) biased assessment tools cannot help us to advance, c) ON research has contributed to pathologising healthy approaches to eating, and d) this area of research needs better psychometric and theoretical approaches for trying to understand this relevant phenomenon. The meta-analysis [1] clearly illustrates that psychometric quality plays a largely neglected role in the ON field. Considering that the problems with the ORTO-15 have been articulated for several years, it is surprising that so much research that uses it is still being published. Thus, we would like to remind researchers of the saying ‘garbage in, garbage out’: Research – whether original studies or meta-analyses – based on invalid measures is invalid.
